# Liver injury indicators and subsequent cancer development among non‐fatty liver population

**DOI:** 10.1002/cam4.5910

**Published:** 2023-04-04

**Authors:** Hiroshi Ito, Takeshi Kimura, Shimbo Takuro, Michiaki Higashitani, Kazuki Yamamoto, Daiki Kobayashi

**Affiliations:** ^1^ Division of Hospital Medicine University of Tsukuba Hospital Ibaraki Japan; ^2^ The Center for Preventive Medicine St. Luke's International Hospital Tokyo Japan; ^3^ Ohta Nishinouchi Hospital Koriyama Japan; ^4^ Department of Cardiology Tokyo Medical University Ibaraki Medical Center Ibaraki Japan; ^5^ Department of Gastroenterology St. Luke's International Hospital Tokyo Japan; ^6^ Division of General Internal Medicine, Department of Medicine Tokyo Medical University Ibaraki Medical Center Ibaraki Japan; ^7^ Fujita Health University Toyoake Japan; ^8^ Department of general medicine Juntendo University Faculty of Medicine Tokyo Japan; ^9^ Division of General Internal Medicine, Department of Medicine St Lukes International Hospital Tokyo Japan

**Keywords:** cancer, fatty liver index, FIB‐4 index, nonalcoholic fatty liver disease fibrosis score

## Abstract

**Background:**

Little is known about the association between liver indicators (The FIB‐4 index, nonalcoholic fatty liver disease fibrosis score (NFS), and fatty liver index (FLI)) and cancer development in patients without preexisting liver disease.

**Methods:**

We conducted a retrospective cohort study with participants who underwent voluntary health checkups and without fatty liver between 2005 and 2018. Our primary outcome was the development of any type of cancer, and its association with each liver indicator was evaluated.

**Results:**

A total of 69,592 participants (mean age: 43.9 years, 29,984 (43.1%) were men) were included. During a median follow‐up of 5.1 years, 3779 (5.4%) patients developed cancer. Compared to participants with a low NFS, those with a medium NFS had a higher risk of developing any type of cancer (adjusted hazard ratio [HR]: 1.18, 95% confidence interval [CI]: 1.07–1.31), whereas those with a medium FIB‐4 index had a decreased risk of developing any type of cancer compared to those with a low FIB‐4 index (adjusted HR: 0.91, 95% CI: 0.83–0.99). Patients with higher scores tended to have a higher risk of digestive organ cancer, regardless of the indicator. A high FLI was also associated with an increased risk of breast cancer (adjusted HR: 2.42, 95% CI: 1.24–4.71); however, those with a medium FIB‐4 index (adjusted HR: 0.65, 95% CI: 0.52–0.81) and NFS (adjusted HR: 0.50, 95% CI: 0.35–0.72) had decreased risks of developing breast cancer compared to those with a high FIB‐4 index and NFS, respectively.

**Conclusion:**

Among patients without fatty liver, a higher liver indicator score was associated with an increased risk of cancer in the digestive organs, regardless of the indicator. Notably, those with a medium FIB‐4 index or NFS had a lower risk of developing breast cancer, whereas those with a medium FLI had an increased risk.

## Graphical abstract

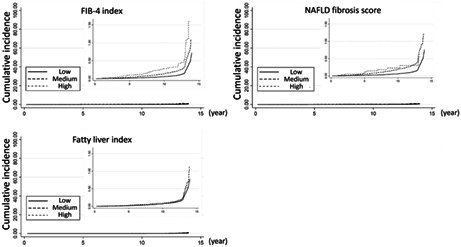

## 1 INTRODUCTION

Liver injury is associated with the development and prognosis of extrahepatic and hepatic cancers. For example, chronic hepatitis C virus infection is a risk factor for pancreatic cancer and non‐Hodgkin lymphoma.^1^ Previous studies have reported that hepatitis B virus infection is positively associated with extrahepatic cancers, such as gastrointestinal cancer, pancreatic cancer, and non‐Hodgkin lymphoma.^2^, ^3^ Furthermore, nonalcoholic fatty liver disease (NAFLD) has been reported to be a risk factor for extrahepatic cancers, such as renal/urological cancer and gastrointestinal cancers other than hepatocellular carcinoma.^4^, ^5^, ^6^ Therefore, liver injury may serve as a risk factor for several types of extrahepatic cancer.^7^

Although liver biopsy is one of the most accurate approaches for evaluating liver injury, is invasive and expensive. Therefore, less invasive indicators for evaluating liver injury have been developed, including the FIB‐4 index, NAFLD fibrosis score (NFS), and fatty liver index (FLI). The FIB‐4 index and NFS were developed as indicators of liver fibrosis in patients with hepatitis C virus infection^8^, ^9^ and NAFLD, respectively.^10^ The FLI was developed as an indicator for hepatic steatosis in the general population.^11^ It has been speculated that indicators of liver injury may predict the development of certain cancers. A higher FIB‐4 index has been reported to be a prognostic factor for several cancer types. For example, a higher FIB‐4 index is associated with increased mortality among patients with hepatocellular carcinoma^12^, ^13^, ^14^ and among those who undergo hepatectomy for colorectal cancer liver metastases.^15^ A higher FIB‐4 index is also associated with anastomotic leakage among patients who undergo surgery for esophageal cancer^16^ and increased mortality among those who undergo surgery for stomach cancer.^17^ These results suggest that some indicators of liver injury may be useful in evaluating cancer prognosis, but few studies have evaluated the usefulness of these indicators for predicting cancer development. Although previous studies have reported that a higher FIB‐4 index and NFS are associated with the development of hepatocellular carcinoma among patients with hepatitis B virus infection, hepatitis C virus infection, alcoholic cirrhosis, and NAFLD,^18^, ^19^, ^20^ few studies have evaluated the association between liver injury indicators and the development of extrahepatic cancer. Our hypothesis is that these liver indicators can predict extrahepatic cancer, because liver injury, including NAFLD, has been reported to be related to cancer development inside and outside the liver. Although previous some previous studies evaluated the association between the degree of liver injury and subsequent cancer development among patients with liver disease, such as NAFLD, very limited studies evaluated the association among patients without liver disease, including fatty liver. In other words, it is unknown whether these indicators are useful for patients without preexisting liver disease because previous studies on these indicators predominantly focused on patients with hepatitis B virus infection, hepatitis C virus infection, alcoholic cirrhosis, or NAFLD.^18^, ^19^, ^20^ Therefore, we aimed to evaluate the association of the FIB‐4 index, NFS, and FLI with subsequent development of any type of cancer among patients without fatty liver.

## 2 METHODS

A retrospective cohort study was conducted at the St. Luke's International Hospital, Tokyo, Japan using the St. Luke's Health Checkup Database. All participants who visited the Center for Preventive Medicine at the hospital underwent voluntary health checkups between 2005 and 2018 were included in the study. We excluded those who had a prior history of any type of cancer during the baseline health checkups. Because we focused only on patients who did not have any liver disease, including fatty liver, as discussed above, those with severe liver disease, such as liver cirrhosis, or fatty liver at baseline based on the findings of abdominal ultrasonography as a part of the health checkups were also excluded. As a part of health checkups, all participants underwent abdominal ultrasounds to evaluate abdominal disease, including fatty liver. Based on the finding in initial abdominal ultrasound, we excluded participants with fatty liver. As a result, scores in each liver fibrosis index including FLI, may be lower than general population. Participants were followed‐up either when they visited to the center for health checkups voluntarily or visited to the hospital for other reasons than health checkups by the end of 2018. The time from baseline date to the last follow‐up date was calculated and used for time variable for survival analyses. Once participants developed any type of cancer, the follow‐ups were terminated at the time of diagnosis. Therefore, there was no reverse causality for the association between liver injury and subsequent cancer development. Our primary outcome was the development of any type of cancer in the follow‐up period. Outcomes were evaluated based on liver fibrosis indexes. The St. Luke's Ethics Committee Institutional Review Board approved this study (approval number: 21‐R165).

### 2.1 Subsequent cancer development

Our primary outcome was the development of any type of cancer during follow‐up, and the secondary outcome was the development of each type of cancer based on the International Classification of Diseases, 10th revision. Information about cancer development was obtained from participants' self‐reports and their electronic medical records at the hospital or the hospital's cancer registry. As part of the health checkups, all participants were asked about their current and past histories, including cancer development, at every visit. In addition, information about cancer diagnoses made by each physician at the hospital was obtained from electronic medical records. Information about cancer diagnoses made in hospitals other than St. Luke's was collected from the cancer registry at the hospitals or referral documents from other hospitals. We have combined this information with our outcomes.

### 2.2 Liver fibrosis indexes

As a part of the health checkups, all participants reported their demographic information (age and sex) and medical history (diabetes), blood tests were performed (liver enzymes [aspartate aminotransferase (AST), alanine aminotransferase (ALT), and γ‐glutamyl transferase (GTP)], serum albumin, triglyceride, and platelets), and height, weight, and abdominal circumference were measured. Body mass index (BMI) was calculated and categorized based on the World Health Organization's Asian criteria, as follows: underweight (<18.5 kg/m^2^), normal weight (>18.5 to ≤24.9 kg/m^2^), and overweight/obese (>24.9 kg/m^2^). Based on this information at baseline, the FIB‐4 index,^21^ NFS,^10^ and FLI^11^ were calculated as follows:

FIB‐4 index = (AST × age)/(platelet count × ALT^0.5^)

NFS = −1.675 + 0.037 × age (years) + 0.094 × BMI (kg/m^2^) + 1.13 × impaired fasting glucose/diabetes (yes = 1, no = 0) + 0.99 × (AST/ALT) − 0.013 × platelet count (×10 ^9^ /L) − 0.66 × albumin (g/dL).

FLI = exp(0.953 × log(triglycerides) + 0.139 × body mass index +0.718 × log(γ‐GTP) + 0.053 × abdominal circumference − 15.745)/(1 + exp(0.953 × log(triglycerides) + 0.139 × BMI + 0.718 × log(γ‐GTP) + 0.053 × abdominal circumference − 15.745)) × 100.

Based on these indexes, we categorized participants into three groups: low (<1.3 for FIB‐4 index, −1.455 for NFS, and 30 for FLI), medium (1.3–2.67 for FIB‐4 index, −1.455–0.675 for NFS, and 30–60 for FLI), and high (>2.67 for FIB‐4 index, 0.675 for NFS, and 60 for FLI).^10^, ^11^, ^22^

### 2.3 Potential confounders

As potential confounders for the association between each index and outcomes, we obtained the participants' demographic information, social histories, and medical histories based on questionnaires administered in the health checkup. Social histories included the participants' smoking status (never, former, or current smoker), alcohol consumption status (abstained, occasional consumption, or regular consumption), and exercise habits (almost none, 1–2 times a week, 3–5 times a week, and almost every day). In addition, medical histories, including diabetes, hypertension, and family history of any type of cancer, were obtained. Models driven by each index were compared using C‐statistics.

### 2.4 Statistical methods

We summarized the participants' characteristics separately based on the score for each index. Then, cumulative incidences for subsequent development of any type of cancer were drawn based on the score (low, medium, or high) for each index, and the log‐rank test was performed to identify significant differences. Finally, the Cox proportional hazards model was applied to evaluate the association between each index and outcomes after adjusting for potential confounders.

All analyses were performed using Stata MP 16.2 in 2022 (STATA Corp.).

## 3 RESULTS

**FIGURE 1:**
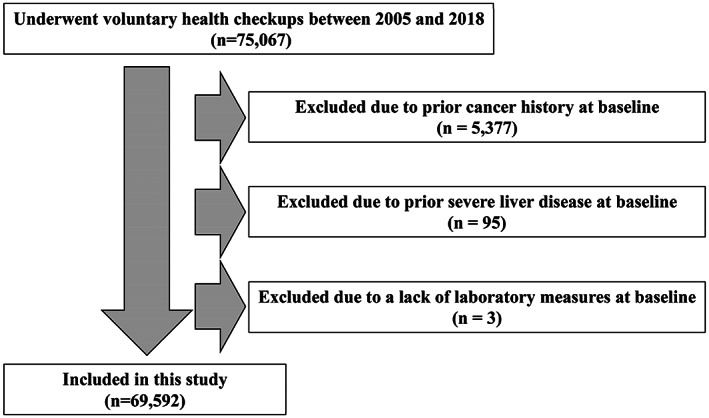
The flow diagram of the study population.

After excluding participants with a prior cancer history (*n* = 5377), those with prior severe liver disease (*n* = 95) and those with a lack of laboratory measures at baseline (*n* = 3), 69,592 participants were included in this study (Figure 1). The mean patient age (standard deviation) was 43.9 (12.1) years, and 29,984 patients (43.1%) were men. Tables 1 and 2 show the participants' baseline characteristics based on the FIB‐4 index, NFS, and FLI. Regardless of the index, participants with higher scores were more likely to be older and male. In terms of social habits, those with higher FIB‐4 index or NFS tended to have healthier lifestyles, whereas those with a higher FLI tended to have unhealthier lifestyles. Participants with higher index scores, regardless of the index, had higher levels of liver enzymes and were more likely to have diabetes and hypertension.

**TABLE 1:** Summary statistics of baseline participants' characteristics among all participants (*n* = 69,592).

	All participants (*n* = 69,592)	
		
Component of FIB‐4 Index		
Age, years, mean (SD)	43.9	(12.1)
Aspartate aminotransferase (AST), IU/L, mean (SD)	20.4	(8.7)
Alanine aminotransferase (ALT), IU/L, mean (SD)	19.0	(11.6)
Platelet, 10^3^/μL, mean (SD)	237.6	(51.2)
Component of NAFLD fibrosis score (age, AST, ALT, platelet were omitted)		
Body mass index, kg/m^2^, mean (SD)	21.5	(2.7)
Diabetes, *n* (%)	812	(1.2)
Albumin, g/dL, mean (SD)	4.4	(0.3)
Component of NAFLD fibrosis score (BMI was omitted)		
γ‐glutamyl transpeptidase (γ‐GTP), IU/L, mean (SD)	28.6	(34.0)
Triglyceride, mg/dL (SD)	82.7	(56.3)
Abdominal circumference, cm, mean (SD)	77.3	(8.1)

**TABLE 2:** Summary statistics of baseline participants' characteristics by score, separately by each index.

	FIB‐4 Index						NAFLD fibrosis score						Fatty liver index					
	Low score		Medium score		High score		Low score		Medium score		High score		Low score		Medium score		High score	
	(*n* = 57,885)		(*n* = 2770)		(*n* = 434)		(*n* = 64,300)		(*n* = 5076)		(*n* = 66)		(*n* = 52,692)		(*n* = 7043)		(*n* = 1961)	
Age, years, mean (SD)	40.7	(9.8)	59.2	(9.7)	67.7	(12.6)	42.4	(10.9)	62.3	(10.8)	70.8	(12.5)	44.7	(11.5)	49.3	(11.6)	47.7	(10.5)
Male, *n* (%)	24,682	(42.6)	5071	(45.0)	231	(53.2)	27,171	(42.3)	2755	(54.3)	43	(65.2)	18,518	(35.1)	5829	(82.8)	1763	(89.9)
Alcohol consumption, *n* (%)																		
Abstainer	22,865	(39.5)	4728	(41.9)	205	(47.2)	25,501	(39.7)	2147	(42.3)	32	(48.5)	21,502	(40.8)	1473	(20.9)	269	(13.7)
Occasional	10,450	(18.1)	1597	(14.2)	56	(12.9)	11,392	(17.7)	692	(13.6)	12	(18.2)	10,088	(19.2)	1002	(14.2)	210	(10.7)
Regular	24,570	(42.5)	4948	(43.9)	173	(39.9)	27,407	(42.6)	2237	(44.1)	22	(33.3)	21,102	(40.1)	4568	(64.9)	1482	(75.6)
Smoking status, *n* (%)																		
Never smoker	37,739	(65.2)	7192	(63.8)	256	(59.0)	42,039	(65.4)	2976	(58.6)	40	(60.6)	36,269	(68.8)	2900	(41.2)	605	(30.9)
Former smoker	10,589	(18.3)	2942	(26.1)	137	(31.6)	12,086	(18.8)	1544	(30.4)	22	(33.3)	9717	(18.4)	2334	(33.1)	697	(35.5)
Current smoker	9557	(16.5)	1139	(10.1)	41	(9.5)	10,175	(15.8)	556	(11.0)	4	(6.1)	6706	(12.7)	1809	(25.7)	659	(33.6)
Exercise habits, *n* (%)																		
Almost none	23,877	(41.3)	2727	(24.2)	116	(26.7)	25,491	(39.6)	1167	(23.0)	22	(33.3)	20,031	(38.0)	2362	(33.5)	710	(36.2)
1–2 times a week	21,663	(37.4)	3961	(35.1)	136	(31.3)	23,975	(37.3)	1704	(33.6)	17	(25.8)	19,335	(36.7)	2882	(40.9)	818	(41.7)
3–5 times a week	7780	(13.4)	2726	(24.2)	105	(24.2)	9292	(14.5)	1274	(25.1)	19	(28.8)	8331	(15.8)	1086	(15.4)	265	(13.5)
Almost everyday	4565	(7.9)	1859	(16.5)	77	(17.7)	5542	(8.6)	931	(18.3)	8	(12.1)	4995	(9.5)	713	(10.1)	168	(8.6)
Body mass index, kg/m^2^, mean (SD)	21.5	(2.7)	21.6	(2.7)	21.4	(3.1)	21.4	(2.7)	22.7	(2.8)	23.8	(3.4)	20.9	(2.3)	24.6	(2.1)	26.6	(2.8)
Abdominal circumference, cm, mean (SD)	77.0	(8.0)	78.9	(8.1)	79.2	(8.8)	76.9	(7.9)	81.9	(8.3)	86.1	(9.1)	75.5	(6.8)	86.9	(5.4)	92.0	(6.9)
Laboratory measures																		
Triglyceride, mg/dL, mean (SD)	0.8	(0.3)	0.8	(0.3)	0.9	(0.3)	0.8	(0.3)	0.8	(0.3)	0.8	(0.3)	0.8	(0.3)	0.8	(0.3)	0.8	(0.3)
AST, IU/L, mean (SD)	19.5	(5.2)	24.1	(8.5)	45.6	(75.1)	20.2	(8.4)	22.9	(10.0)	34.9	(54.3)	19.9	(7.3)	23.3	(8.6)	27.0	(10.9)
ALT, IU/L, mean (SD)	18.8	(10.6)	19.9	(11.4)	32.1	(53.5)	19.1	(11.7)	18.0	(9.9)	18.4	(13.8)	17.4	(9.1)	26.3	(14.4)	34.4	(18.5)
γ‐GTP, IU/L, mean (SD)	27.6	(31.1)	32.8	(40.1)	52.9	(112.0)	28.4	(33.6)	30.9	(36.3)	47.4	(107.9)	21.8	(15.9)	58.3	(49.1)	110.6	(97.4)
Albumin, g/dL, mean (SD)	4.4	(0.2)	4.4	(0.2)	4.3	(0.3)	4.4	(0.2)	4.3	(0.2)	4.2	(0.3)	4.4	(0.2)	4.5	(0.2)	4.5	(0.2)
Platelet, 10^3^/μl, mean (SD)	245.9	(49.8)	198.1	(34.7)	151.8	(41.4)	241.9	(49.9)	184.5	(34.5)	151.4	(46.2)	236.0	(51.1)	241.8	(51.4)	245.7	(56.5)
Hypertension, *n* (%)	1785	(3.1)	1600	(14.2)	116	(26.7)	2435	(3.8)	1029	(20.3)	23	(34.9)	2157	(4.1)	906	(12.9)	288	(14.7)
Diabetes, *n* (%)	398	(0.7)	387	(3.4)	27	(6.2)	214	(0.3)	559	(11.0)	37	(56.1)	515	(1.0)	194	(2.8)	59	(3.0)
Family history of cancer, *n* (%)	22,311	(38.5)	5210	(46.2)	184	(42.4)	25,353	(39.4)	2265	(44.6)	22	(33.3)	22,045	(41.8)	2777	(39.4)	733	(37.4)
Viral hepatitis																		
Positive for HCV antibody	2388	(4.2)	537	(4.9)	45	(10.5)	2658	(4.2)	308	(6.2)	4	(6.2)	2605	(5.1)	272	(3.9)	76	(3.9)
Positive for HBs antigen	312	(0.6)	171	(1.5)	15	(3.5)	420	(0.7)	78	(1.6)	0	(0.0)	391	(0.8)	58	(0.8)	17	(0.9)
Positive for HBs antibody	6916	(12.3)	1888	(17.0)	96	(22.5)	7927	(12.7)	936	(18.7)	15	(23.1)	5632	(11.0)	988	(14.3)	264	(13.7)

Abbreviations: AST, aspartate aminotransferase; ALT, alanine aminotransferase; γ‐GTP, γ‐glutamyl transpeptidase.

**FIGURE 2:**
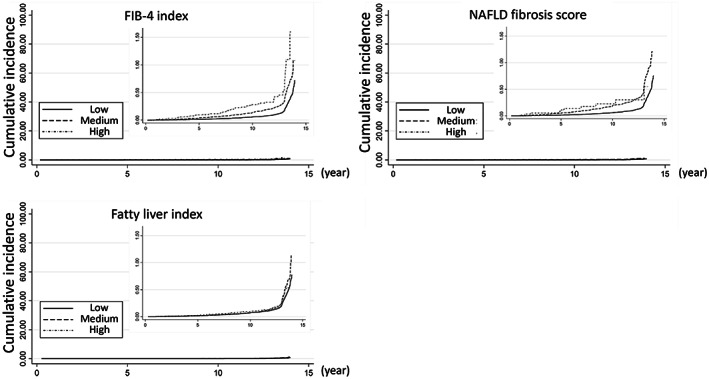
Cumulative incidence of any type of cancer by scores in each indicator.

A median duration of follow‐up of the participants was 5.1 years (interquartile range: 2.1–9.7 years) and a median number of follow‐ups was 5 times (interquartile range: 3–11 times). During the period, 3779 (5.4%) patients developed any type of cancer. Figure 2 shows the cumulative incidences for the development of any type of cancer based on the index score groups for each index. The differences of cumulative incidence based on the FIB‐4 index were the most obvious, but all indexes were significantly associated with cancer development (all *p* < 0.01). Table 3 shows the results of the Cox proportional hazard model for the development of any type of cancer based on each index adjusted for potential confounders. Compared to participants with low FIB‐4 index, those with medium FIB‐4 index had a lower risk of developing any type of cancer (adjusted hazard ratio [HR] 0.91, 95% confidence interval [CI]: 0.83–0.99). Participants with high NFS and FLI tended to have higher risk for the development of any type of cancer compared to participants with a low NFS and FLI, but only those with medium NFS had a significantly increased risk (adjusted HR 1.18, 95% CI: 1.07–1.31). The model assessing NFS had the statistically highest C‐statistics. We also provided the results of the Cox proportional hazard model for potential confounders (supplement).

**TABLE 3:** Adjusted hazard ratios for the development of any type of cancer by scoring systems and their model comparisons among non‐fatty liver participants.

	Crude hazard ratio (95% confidence interval)			*p* Value for crude hazard ratio		
	Adjusted hazard ratio (95% confidence interval)			*p* Value for adjusted hazard ratio		
	FIB‐4 index		NAFLD fibrosis score		Fatty liver index	
Risk category						
Low score	Reference		Reference		Reference	
	(Cancer cases: 2551) (Follow‐up: 339,801 person‐years)		(Cancer cases: 3126) (Follow‐up: 380,987 person‐years)		(Cancer cases: 2979) (Follow‐up: 324,865 person‐years)	
Medium score	**2.21 (2.06–2.37)**	**<0.01**	**2.72 (2.50–2.96)**	**<0.01**	**1.23 (1.12–1.35)**	**<0.01**
	**0.91 (0.83–0.99)**	**0.03**	**1.18 (1.07–1.31)**	**<0.01**	1.01 (0.90–1.12)	0.92
	(Cancer cases: 1157) (Follow‐up: 70,117 person‐years)		(Cancer cases: 634) (Follow‐up: 30,322 person‐years)		(Cancer cases: 519) (Follow‐up: 44,112 person‐years)	
High score	**4.45 (3.52–5.64)**	**<0.01**	**3.56 (1.92–6.63)**	**<0.01**	**1.31 (1.11–1.54)**	**<0.01**
	1.22 (0.95–1.57)	0.13	1.47 (0.78–2.78)	0.24	1.20 (0.99–1.45)	0.06
	(Cancer cases: 71) (Follow‐up: 2420 person‐years)		(Cancer cases: 10) (Follow‐up: 389 person‐years)		(Cancer cases: 150) (Follow‐up: 12,082 person‐years)	
Model comparison	β coefficient	*p* value	β coefficient	*p* value	β coefficient	*p* value
vs. FIB‐4 Index	—		**0.00092**	**0.04**	−0.000609	0.17
vs. NAFLD fibrosis score	**−0.00092**	**0.04**	—		**−0.001532**	**<0.01**
vs. Fatty liver index	0.000609	0.17	**0.001532**	**<0.01**	—	

Models were adjusted for age, gender, body mass index, smoking status, alcohol consumption, exercise habits, medical history of hypertension, diabetes, family history of any type of cancer, HCV antibody, HBs antigen and HBs antibody.

The numbers in bold represent that the *p*‐value is <0.05.

Table 4 shows a comparison of the models with different indexes for the development of each type of cancer. Participants with high FIB‐4 index had an increased risk of developing cancer in the lips, oral cavity, and pharynx (C00–C14) than those with low FIB‐4 index (adjusted HR 15.9, 95% CI: 2.06–123.00). For cancers in the digestive organs (C15–C26), including hepatocellular carcinoma, medium scores of all three indexes were associated with an increased risk compared to low scores, and high scores were associated with an even further increased risk. Interestingly, participants with a medium FIB‐4 index or NFS had lower adjusted HRs for the development of breast cancer (0.65, 95% CI: 0.52–0.81 for the FIB‐4 index and 0.50, 95% CI: 0.35–0.72 for the NFS) than those with a low FIB‐4 index and NFS, respectively, but those with high FLI had a higher adjusted HR for the development of breast cancer (2.42, 95% CI: 1.24–4.71).

**TABLE 4:** Adjusted hazard ratios for the development of each type of cancer and model comparison between scores among participants without fatty liver.

	Adjusted hazard ratio (95% confidence interval)					
	FIB‐4 Index		NAFLD fibrosis score		Fatty liver index	
C00–C14: Lips, oral cavity and pharynx (*n* = 13)						
Low score	Reference		Reference		Reference	
Medium score	0.56	(0.10–3.25)	2.26	(0.48–10.7)	0.32	(0.03–2.97)
High score	**15.9**	**(2.06–123)**	—^a^		1.18	(0.09–14.8)
Model comparison	β coefficient	p value	β coefficient	*p* value	β coefficient	*p* value
vs. FIB‐4 Index	—		0.02134	0.61	0.01926	0.83
vs. NAFLD fibrosis score	−0.02134	0.61	—		−0.002087	0.97
vs. Fatty liver index	−0.01926	0.83	0.002087	0.97	—	
C15–C26: Digestive organs (*n* = 989)						
Low score	Reference		Reference		Reference	
Medium score	1.00	(0.85–1.18)	1.19	(0.99–1.42)	1.11	(0.93–1.34)
High score	**1.69**	**(1.17–2.46)**	1.29	(0.47–3.58)	**1.46**	**(1.08–1.97)**
Model comparison	β coefficient	p value	β coefficient	p value	β coefficient	*p* value
vs. FIB‐4 Index	—		0.00076	0.07	−0.0000796	0.92
vs. NAFLD fibrosis score	−0.00076	0.07	—		−0.000838	0.28
vs. Fatty liver index	0.0000796	0.92	0.000838	0.28	—	
Hepatocellular carcinoma (*n* = 20)						
Low score	Reference		Reference		Reference	
Medium score	1.31	(0.38–4.48)	3.00	(0.93–9.71)	1.54	(0.46–5.14)
High score	**14.7**	**(3.13–68.6)**	11.68	(0.89–154)	1.68	(0.26–11.0)
Model comparison	β coefficient	*p* value	β coefficient	*p* value	β coefficient	*p* value
vs. FIB‐4 Index	—		−0.00266	0.22	−0.0004235	0.94
vs. NAFLD fibrosis score	0.00266	0.22	—		0.00224	0.69
vs. Fatty liver index	0.0004235	0.94	−0.00224	0.69	—	
Other digestive organs than liver (*n* = 970)						
Low score	Reference		Reference		Reference	
Medium score	0.99	(0.84–1.17)	1.17	(0.97–1.40)	1.11	(0.92–1.34)
High score	1.48	(0.99–2.20)	1.04	(0.32–3.32)	**1.47**	**(1.08–1.99)**
Model comparison	β coefficient	p value	β coefficient	p value	β coefficient	*p* value
vs. FIB‐4 Index	—		0.00068	0.08	−0.0002645	0.72
vs. NAFLD fibrosis score	−0.00068	0.08	—		−0.0009445	0.23
vs. Fatty liver index	0.0002645	0.72	0.0009445	0.23	—	
C30–C39: Respiratory and intrathoracic organs (*n* = 353)						
Low score	Reference		Reference		Reference	
Medium score	0.91	(0.70–1.19)	1.01	(0.76–1.36)	0.89	(0.63–1.25)
High score	0.73	(0.33–1.62)	0.78	(0.10–5.82)	1.22	(0.67–2.22)
Model comparison	β coefficient	*p* value	β coefficient	*p* value	β coefficient	*p* value
vs. FIB‐4 Index	—		0.00033	0.48	−0.0009487	0.38
vs. NAFLD fibrosis score	−0.00033	0.48	—		−0.001283	0.18
vs. Fatty liver index	0.0009487	0.38	0.001283	0.18	—	
C40–C41: Bone and articular cartilage (*n* = 1)	— ^a^					
C43–C44: Melanoma and other skin (*n* = 27)						
Low score	Reference		Reference		Reference	
Medium score	1.73	(0.63–4.75)	0.61	(0.16–2.33)	—	
High score	—^a^		—^a^		1.10	(0.17—6.99)
Model comparison	β coefficient	*p* value	β coefficient	*p* value	β coefficient	*p* value
vs. FIB‐4 Index	—		0.04999	0.33	0.0668893	0.23
vs. NAFLD fibrosis score	−0.04999	0.33	—		0.0169	0.52
vs. Fatty liver index	−0.0668893	0.23	−0.0169	0.52	—	
C45–C49: Mesothelial and soft tissue (*n* = 7)						
Low score	Reference		Reference		Reference	
Medium score	0.68	(0.09–5.04)	10.6	(1.55–72.4)	—^a^	
High score	—^a^		—^a^		—^a^	
Model comparison	β coefficient	*p* value	β coefficient	*p* value	β coefficient	*p* value
vs. FIB‐4 Index	—		0.053	0.01	—^a^	
vs. NAFLD fibrosis score	−0.053	0.01	—		—^a^	
vs. Fatty liver index	—^a^		—^a^		—	
C50–C50: Breast tissue (*n* = 910)						
Low score	Reference		Reference		Reference	
Medium score	**0.65**	**(0.52–0.81)**	**0.50**	**(0.35–0.72)**	1.04	(0.7–1.53)
High score	0.49	(0.15–1.53)	—^a^		**2.42**	**(1.24–4.71)**
Model comparison	β coefficient	*p* value	β coefficient	*p* value	β coefficient	*p* value
vs. FIB‐4 Index	—		−0.004607	0.19	−0.0075737	0.08
vs. NAFLD fibrosis score	0.004607	0.19	—		−0.002967	0.48
vs. Fatty liver index	0.0075737	0.08	0.002967	0.48	—	
C51–C58: Female genital organs (*n* = 419)						
Low score	Reference		Reference		Reference	
Medium score	0.85	(0.62–1.17)	1.36	(0.9–2.06)	1.25	(0.72–2.17)
High score	1.48	(0.46–4.76)	5.65	(0.78–41.1)	0.49	(0.07–3.63)
Model comparison	β coefficient	p value	β coefficient	p value	β coefficient	*p* value
vs. FIB‐4 Index	—		0.00037	0.94	0.0004594	0.93
vs. NAFLD fibrosis score	−0.00037	0.94	—		0.0000868	0.99
vs. Fatty liver index	−0.0004594	0.93	−0.0000868	0.99	—	
C60–C63: Male genital organs (*n* = 499)						
Low score	Reference		Reference		Reference	
Medium score	0.85	(0.68–1.07)	0.79	(0.63–1)	1.02	(0.81–1.27)
High score	0.55	(0.3–1.01)	0.46	(0.14–1.49)	0.97	(0.63–1.49)
Model comparison	β coefficient	*p* value	β coefficient	*p* value	β coefficient	*p* value
vs. FIB‐4 index	—		0.0000311	0.91	0.0002598	0.39
vs. NAFLD fibrosis score	−0.0000311	0.91	—		0.0002288	0.44
vs. Fatty liver index	−0.0002598	0.39	−0.0002288	0.44	—	
C64–C68: Urinary tract (*n* = 124)						
Low score	Reference		Reference		Reference	
Medium score	0.89	(0.57–1.4)	1.65	(1.02–2.66)	1.05	(0.63–1.76)
High score	—^a^		—^a^		1.20	(0.49–2.91)
Model comparison	β coefficient	*p* value	β coefficient	*p* value	β coefficient	*p* value
vs. FIB‐4 Index	—		0.0126922	0.42	0.0126545	0.42
vs. NAFLD fibrosis score	−0.0126922	0.42	—		−0.0000377	0.99
vs. Fatty liver index	−0.0126545	0.42	0.0000377	0.99	—	
C69–C72: Eye, brain and other parts of central nervous system (*n* = 2)						
Low score	Reference		Reference		Reference	
Medium score	0.45	(0.01–15.6)	1.45	(0.04–51.2)	2.03	(0.07–57.4)
High score	—^a^		—^a^		—^a^	
Model comparison	β coefficient	p value	β coefficient	p value	β coefficient	*p* value
vs. FIB‐4 Index	—		−0.0003907	0.90	**0.0016324**	**<0.01**
vs. NAFLD fibrosis score	0.0003907	0.90	—		0.002023	0.56
vs. Fatty liver index	**−0.0016324**	**<0.01**	−0.002023	0.56	—	
C73–C75: Thyroid and other endocrine glands (*n* = 33)						
Low score	Reference		Reference		Reference	
Medium score	1.12	(0.38–3.28)	0.82	(0.18–3.78)	1.66	(0.44–6.36)
High score	7.12	(0.81–63.0)	—^a^		1.95	(0.19–20.1)
Model comparison	β coefficient	*p* value	β coefficient	*p* value	β coefficient	*p* value
vs. FIB‐4 Index	—		0.0079554	0.12	**0.0204618**	**<0.05**
vs. NAFLD fibrosis score	−0.0079554	0.12	—		0.0125064	0.17
vs. Fatty liver index	**−0.0204618**	**<0.05**	−0.0125064	0.17	—	
C76–C80: Ill‐defined, other secondary and unspecified sites (*n* = 33) —^a^						
C81–C96: Lymphoid, hematopoietic and related tissues (*n* = 84)						
Low score	Reference		Reference		Reference	
Medium score	0.66	(0.36–1.20)	0.99	(0.49–1.97)	0.78	(0.36–1.72)
High score	0.64	(0.08–5.02)	—^a^		1.54	(0.47–5.07)
Model comparison	β coefficient	*p* value	β coefficient	*p* value	β coefficient	*p* value
vs. FIB‐4 Index	—		0.0034532	0.52	0.0080895	0.36
vs. NAFLD fibrosis score	−0.0034532	0.52	—		0.0046363	0.52
vs. Fatty liver index	−0.0080895	0.36	−0.0046363	0.52	—	
D00‐D09: In situ neoplasms (*n* = 75)						
Low score	Reference		Reference		Reference	
Medium score	1.10	(0.48–2.51)	1.09	(0.32–3.69)	0.63	(0.17–2.37)
High score	—^a^		—^a^		—^a^	
Model comparison	β coefficient	*p* value	β coefficient	*p* value	β coefficient	*p* value
vs. FIB‐4 Index	—		−0.0007408	0.44	−0.0087782	0.66
vs. NAFLD fibrosis score	0.0007408	0.44	—		−0.0080374	0.69
vs. Fatty liver index	0.0087782	0.66	0.0080374	0.69	—	
Unknown (*n* = 238)						
Low score	Reference		Reference		Reference	
Medium score	0.93	(0.65–1.31)	1.38	(0.93–2.04)	1.07	(0.71–1.62)
High score	1.38	(0.53–3.57)	3.00	(0.67–13.5)	1.42	(0.74–2.73)
Model comparison	β coefficient	*p* value	β coefficient	*p* value	β coefficient	*p* value
vs. FIB‐4 Index	—		0.0015125	0.48	0.0035999	0.22
vs. NAFLD fibrosis score	−0.0015125	0.48	—		0.0020874	0.55
vs. Fatty liver index	−0.0035999	0.22	−0.0020874	0.55	—	

Models were adjusted for age, gender, body mass index, smoking status, alcohol consumption, exercise habits, medical history of hypertension, diabetes and family history of any type of cancer, HCV antibody, HBs antigen, and HBs antibody.

The numbers in bold represent that the *p*‐value is <0.05.

^a^ Hazard ratios could not be calculated due to insufficient number of events.

## 4 DISCUSSION

Participants with medium NFS tended to have a higher risk of developing any type of cancer than those with low NFS. By contrast, patients with medium FIB‐4 index tended to have a lower risk of developing any type of cancer than those with low FIB‐4 index. With regard to specific cancer types, patients with high FIB‐4 index had a significantly higher risk of developing oropharyngeal and digestive organ cancers, including hepatocellular carcinoma. A high FLI was also associated with the development of digestive organ cancer. Notably, participants with high FLI had higher risk of developing breast cancer; however, those with medium FIB‐4 index or NFS had lower risk of developing breast cancer.

Our results partially correspond to those of previous studies, even though our target population, individuals without fatty liver, and target cancer types were different from those of previous studies. For example, the FIB‐4 index has been reported to be related to hepatocellular cancer development in patients with liver disease, such as those with chronic hepatitis C virus infection without sustained virological response after interferon‐based therapy,^18^ alcoholic liver cirrhosis,^19^ or coexisting NAFLD and chronic hepatitis B.^20^ We found that a high FIB‐4 index was associated with an increased risk of hepatocellular carcinoma among individuals without fatty liver. Several previous studies have shown that a high FLI is associated with increased risks of colorectal adenoma among the general population,^23^ colorectal cancer among people without obesity or other metabolic syndromes,^24^ and breast cancer development among postmenopausal women.^25^ These results are similar to our finding that high FLI is associated with an increased risk of cancer in the digestive organs and breast cancer. Therefore, our results suggest that the FIB‐4 index and FLI are useful for predicting the development of certain types of cancer, even among individuals without fatty liver. Similarly, we observed an association between the severity of fibrosis as scored by the NFS and the development of any type of cancer or hepatocellular carcinoma in our population, although some associations were not statistically significant. Similar results have also been reported among NAFLD patients in previous studies.^26^, ^27^

We hypothesized that the differences we observed in the direction and magnitude of the associations between the score for each indicator and the development of any or a certain type of cancer may result from differences in the origin and purpose of the indicators. The FIB‐4 index and NFS mainly include hepatobiliary markers, such as AST, ALT, and platelet count, and thus reflect the degree of liver fibrosis. When the liver is injured, apoptotic or necrotic hepatocytes, activated Kupffer cells, hepatic stellate cells (HSCs), and neutrophils produce reactive oxygen species.^28^ This oxidative stress facilitates the activation and migration of HSCs, resulting in liver fibrosis. Hence, a high FIB‐4 index and NFS may reflect excessive oxidative stress, which may be associated with future cancer development.^29^ Conversely, the FLI includes metabolic syndrome‐related variables, such as BMI, abdominal circumference, and triglycerides. It has been proposed that the combination of obesity, inflammation, and insulin resistance is associated with cancer development in patients with metabolic syndrome. Hyperinsulinemia in metabolic syndrome is considered to stimulate cancer cell proliferation through the p21 Ras/mitogen‐activated protein kinase and phosphatidylinositol‐3 kinase/Akt pathways through the IGF‐1 receptor, which is overexpressed in colon cancer.^30^ Hence, it is plausible that a high FLI, a surrogate marker of metabolic syndrome, may be associated with subsequent cancer development.

Our study found that a high FLI was associated with the development of breast cancer; by contrast, a medium FIB‐4 index and NFS were associated with a lower risk of developing breast cancer. Although the underlying mechanism for this finding is unclear, estrogen levels might play a role. Several studies have suggested an association between estrogen deficiency and liver fibrosis, which can be evaluated using the FIB‐4 index or NFS. For example, among patients with chronic hepatitis C virus genotype 1b infection treated with interferon‐based therapy, those with biopsy‐confirmed liver fibrosis had lower estrogen receptor alpha expression than those without fibrosis,^31^ suggesting that advanced liver fibrosis is associated with decreased estrogen signaling. Among women with untreated chronic hepatitis B infection, liver stiffness measurements using transient elastography indicate that menopause and late menarche aggravate liver fibrosis, suggesting an effect of estrogen deficiency on liver fibrosis progression.^32^ In an experimental study using CCL4‐induced liver fibrosis in rats, estrogen reduced serum AST, ALT, hyaluronic acid, and type IV collagen, suppressed hepatic collagen content, decreased the percentage of HSCs positive for α‐smooth muscle actin, and significantly lowered the synthesis of hepatic type I collagen.^33^ Because high estrogen levels are an established risk factor for breast cancer development,^34^ we speculated that individuals with a mildly elevated FIB‐4 index or NFS had decreased levels of estrogen and thus had a lower risk of developing breast cancer. In contrast, the findings that a high FLI was associated with breast cancer development may be explained by estrogen exposure from the adipose tissue.^35^ Obesity is a known risk factor for developing breast cancer,^36^ and BMI is positively correlated with estrogen levels in the tissue.^37^, ^38^ Because our analysis was adjusted for BMI, the association between a high FLI and a high incidence of breast cancer may reflect not only obesity but also high abdominal circumference, a surrogate marker of large‐volume visceral adipose tissue.^39^ As fat mass increases, estrogen levels also increase. Elevated estrogen levels have a pro‐proliferative effect on the breast epithelium, which may cause the accumulation of replication errors, leading to mutations. Through these processes, elevated estrogen levels play an important role in the development of breast cancer.

This study had several strengths. First, it included a large population of approximately 70,000 participants. This large sample size provided sufficient power for analysis. In addition, our study considered several indicators, including the FIB‐4 index, NFS, and FLI. This information would be useful for examining the mechanisms underlying the study results.

However, our study also has several limitations. First, our study included patients without fatty liver who had relatively low liver fibrosis indexes. The number of participants with high indexes in our study was relatively small, which potentially reduced the statistical power in the high score category. In this context, different cutoff values of these indicators may be considered for individuals without fatty liver to more accurately evaluate the association between the score category of each indicator and cancer development. Second, some participants may have had existing occult cancer at baseline. However, based on the cumulative incidence of cancer, the number of patients with undiagnosed cancer at baseline was limited. Third, our exclusion criterion regarding fatty liver was based on the findings of abdominal ultrasonography, although the definitive diagnosis of fatty liver requires liver biopsy.^40^ Fourth, we did not have information on essential potential confounders such as prescription of lipid‐lowering drugs and hormone replacement therapy, which may influence the development of liver injury and some types of cancer. This lack of data may have biased the results. Furthermore, our study population may have had higher health awareness than the general population. Additionally, because approximately 70% of the participants were employed, they may have had higher socioeconomic status than those who are unemployed. In addition, our study was performed in Japan and thus included predominantly Japanese people, who have a higher prevalence of lean NAFLD than Caucasian individuals. Therefore, our results may not be generalizable to Western populations. Finally, although our study included large healthy population, the number of patients who developed certain cancers (e.g., lips, oral cavity, and pharynx) was limited due to its very low incidence rate. Therefore, it may be difficult to evaluate the association or interpret the negative associations in certain cancers. Further studies are needed to have more accurate evidence.

## 5 CONCLUSION

Among individuals without fatty liver, those with a medium NFS had a higher risk of developing any type of cancer, whereas those with a medium FIB‐4 index had a lower risk of cancer development. With regard to development of individual cancer types, a higher score was associated with the development of cancer in the digestive organs, regardless of the indicator. However, individuals with a medium FIB‐4 index and NFS had a lower risk of breast cancer, whereas those with a high FLI had an increased risk of developing breast cancer.

## AUTHOR CONTRIBUTIONS

**Hiroshi Ito:** Conceptualization (supporting); writing – original draft (equal); writing – review and editing (supporting). **Takeshi Kimura:** Methodology (supporting); supervision (supporting); writing – review and editing (supporting). **Takuro Shimbo:** Methodology (supporting); supervision (supporting); writing – review and editing (supporting). **Michiaki Higashitani:** Conceptualization (supporting); project administration (supporting); writing – review and editing (supporting). **Kazuki Yamamoto:** Supervision (supporting); writing – review and editing (supporting). **Daiki Kobayashi:** Conceptualization (lead); data curation (lead); formal analysis (lead); investigation (lead); methodology (lead); project administration (lead); resources (lead); software (lead); validation (lead); visualization (lead); writing – review and editing (lead).

## FUNDING INFORMATION

None.

## CONFLICT OF INTEREST STATEMENT

None.

## Data Availability

Data Availability StatementDue to the nature of this research, participants’ data was not shared publicly due to participants’ privacy, so supporting data is not available.
